# Welsh Primary Schoolchildren’s Perceptions of Electronic Cigarettes: A Mixed Methods Study

**DOI:** 10.3390/ijerph17103639

**Published:** 2020-05-21

**Authors:** Lorna Porcellato, Kim Ross-Houle, Zara Quigg, Jane Harris, Charlotte Bigland, Rebecca Bates, Hannah Timpson, Ivan Gee, Julie Bishop, Ashley Gould, Alisha R. Davies

**Affiliations:** 1 Public Health Institute; Faculty of Health, Liverpool John Moores University, Liverpool L2 2QP, UK; Z.A.Quigg@ljmu.ac.uk (Z.Q.); J.Harris@ljmu.ac.uk (J.H.); C.Bigland@ljmu.ac.uk (C.B.); R.Bates@ljmu.ac.uk (R.B.); H.Timpson@ljmu.ac.uk (H.T.); I.L.Gee@ljmu.ac.uk (I.G.); 2 Faculty of Social Sciences, Department of Social and Political Science, University of Chester, Chester CH1 4BJ, UK; k.rosshoule@chester.ac.uk; 3 Public Health Wales, Cardiff CF10 4BZ, UK; julie.bishop4@wales.nhs.uk (J.B.); ashley.gould@wales.nhs.uk (A.G.); Alisha.Davies@wales.nhs.uk (A.R.D.)

**Keywords:** vaping, smoking, e-cigarettes, primary schoolchildren, perceptions, mixed methods, health education

## Abstract

There are concerns that the growing popularity of e-cigarettes promotes experimentation among children. Given the influence of the early years on attitude and habit formation, better understanding of how younger children perceive vaping before experimentation begins is needed, to prevent uptake and inform tobacco control strategies. We explored Welsh primary schoolchildren’s (aged 7–11) awareness of e-cigarettes relative to tobacco smoking, their understanding of the perceived risks and benefits and their intentions and beliefs about vaping. Data was collected using a mix of methods in June and July 2017 from 8 purposively selected primary schools across Wales. Four hundred and ninety-five children (52% female) aged 7 years (*n* = 165), 9 years (*n* = 185) and 11 years (*n* = 145) completed a class-administered booklet encompassing a draw and write exercise and survey. Ninety-six children participated in 24 peer discussion groups comprised of 2 boys and 2 girls from each year group. Data were analysed independently and findings triangulated. Survey analyses used frequencies, descriptive statistics and chi-squared tests. Content analysis was undertaken on the draw and write data and peer discussion groups were analysed thematically. Study findings highlight that primary schoolchildren have general awareness of e-cigarettes. Vaping was perceived to be healthier than smoking and there was some recognition that e-cigarettes were used for smoking cessation. Understanding of any health harms was limited. Few children intended to smoke or vape in the future but almost half thought it was okay for grownups. Children’s perceptions were influenced by exposure through family and friends. Findings suggest a need for e-cigarette education in primary schools, to highlight the associated risks of e-cigarette experimentation including the potential for tobacco initiation.

## 1. Introduction

There has been a global increase in the popularity of e-cigarettes amongst young people [1]. In Great Britain (GB), 15.4% of adolescents aged 11–18 years tried e-cigarettes in 2019, an increase from 12.7% in 2015 [2]. Although some reports suggest that regular use (once a week or more) amongst youth is rare and largely limited to current or previous smokers [2,3,4], awareness of and experimentation with e-cigarettes is rising [2,5,6,7,8]. Similar trends have been noted in other countries including the USA and Canada [9,10].

The escalation of youth e-cigarette use has raised significant concerns, mainly around their potential to act as a gateway to tobacco smoking and the renormalisation of smoking behaviour [6,11,12]. There are also concerns that adolescent e-cigarette use will promote experimentation amongst younger children. Research suggests that experimental ‘ever use’ of e-cigarettes may be linked to uptake of tobacco smoking [13]. In a cross-sectional survey with Welsh primary school children (aged 10–11 years), e-cigarette use was found to be more common than tobacco use [14]. Similarly, findings from the National Youth Tobacco Surveys (NYTS) show that in 2018, e-cigarettes were the most commonly used tobacco product (4.9%; 570,000) among U.S. middle schoolchildren (ages 11–13) [15]. 

Questions about the harms and benefits of using e-cigarettes have also emerged and despite a burgeoning research effort, findings are inconclusive. There is some evidence to suggest the risk to health in the short term is considerably less for e-cigarettes relative to tobacco cigarettes [16,17,18] but the extent to which e-cigarettes are ‘less harmful’ is contested [19]. Moreover, the long-term health effects of vaping are as yet unknown [18]. Opinions on the merits of e-cigarettes as a smoking cessation aid are also divided and data are conflicting [17,20]. Some consider e-cigarettes to be a safer alternative to tobacco cigarettes and promote them as a useful tool to quit smoking [16,17]. Whilst recent research has demonstrated some efficacy [21] and health advantages to vaping relative to smoking [22] evidence remains limited with randomized controlled trials and cohort studies demonstrating insignificant effects [18,23,24].

Despite intense debate and controversy around the use of e-cigarettes, there is a general consensus that they are not risk free and of no benefit to young never smokers [25,26,27]. Most e-cigarettes contain nicotine, a highly addictive substance. Children’s brain development may be ‘vulnerable’ to the negative consequences of nicotine exposure [25,26]. Children are also at risk of nicotine poisoning through accidental ingestion of liquid nicotine [28,29].

Efforts to minimise risk and protect children from exposure to and uptake of e-cigarettes are critical. Currently 100 countries have national laws regulating e-cigarettes [30] although regulation varies widely between them [31]. The UK and domestic regulation enforces an age of sale lower limit of 18 years, prohibits proxy purchasing and the promotion of e-cigarettes on television, radio, online and in some print media. There are also limits on the concentration of nicotine in e-liquids, a requirement for warning labels on products and child tamper proof packaging [32]. 

The exponential growth of youth e-cigarette use has led to calls by some for further regulation. In 2015/16 the Welsh government proposed to ban the use of e-cigarettes in enclosed public places in Wales; the proposal was rejected by the Welsh Assembly. In 2018 the Forum of International Respiratory Societies recommended a worldwide ban on the sale of e-cigarettes to young people, the prohibition of flavourings and use of e-cigarettes where children are present [25]. Recently the U.S Food and Drug Administration (FDA) issued a policy prioritizing enforcement against certain unauthorized flavored e-cigarette products such as fruit and mint flavours that appeal to children [33].

To inform development of current tobacco control policies and prevention strategies, as well as the wider debate on further regulation of e-cigarettes, research on children perspectives of e-cigarettes is needed. Without knowing ‘the extent of each child’s knowledge and understanding’, work may be irrelevant and the important health messages may have little impact [34,35]. Whilst qualitative research has been conducted with adolescents [36,37,38,39], studies that focus on schoolchildren’s perspectives of e-cigarettes are limited and commentary on this issue is hindered by the lack of empirical evidence [6,11,12]. To date only Faletau et al [40] have explored perceptions of e-cigarettes exclusively within this age group. They found 6–10-year-old Maori and Pacific Islander children in New Zealand were unfamiliar with the product and at first glance could not discern the difference between electronic and tobacco cigarettes. Given the pervasiveness of e-cigarettes today, such findings are unlikely to be relevant. 

Evidence from the wider substance misuse literature suggests that children assimilate knowledge about addictive substances before they reach the age of experimentation [41,42,43]. It is widely accepted that tobacco smoking patterns begin prior to experimentation with the development of attitudes and beliefs which may influence future behaviour. Given that young children develop complex understandings of cigarette smoking which may predispose them to start smoking when older [41,42,43], it can be theorised that with the escalation of e-cigarette use, young children may develop equally complex understandings of e-cigarettes that may predispose them to vape in the future as well.

In view of the recognised influence of the early years on attitude and habit formation and the increasing prevalence of e-cigarettes use, better understanding of how young children perceive vaping is needed, given the possible lifelong health harms caused by behaviours established in childhood. This small scale exploratory mixed methods study aimed to explore Welsh primary schoolchildren’s (aged 7–11) awareness of e-cigarettes relative to tobacco smoking, their understanding of the perceived risks and benefits associated with e-cigarettes and their intentions and beliefs about vaping, with a view to informing development of effective prevention strategies and policy responses around e-cigarette use. 

## 2. Materials and Methods 

### 2.1. Sampling and Recruitment

Primary schools located across 8 areas in Wales (*n* = 339) with varying levels of deprivation, prevalence of Welsh language spoken, urban/rural classification and prevalence of adult e-cigarette use were identified for recruitment; single sex, and fee-paying schools were excluded. Selection was purposive and based on maximum variation sampling, to better understand how children living in different parts of Wales, in different contexts perceived vaping. One Welsh speaking school was recruited as well as one school from an area with a high Black, Asian and ethnic minority (BAME) population. The remaining six schools were representative of varying levels of deprivation and geographic spread. We also sought to target schools in areas which had higher levels of adult e-cigarette use identified through the Welsh Health Survey [44]. 

Schools were initially contacted by the Welsh Healthy Schools Co-ordinator to inform them about the project and then invited via a letter from the research team which provided information about the study, the protocol for gaining consent/assent and contact details. Follow up emails and telephone calls were undertaken to determine interest. Of the schools willing to take part (*n* = 12), one from each area (*n* = 8) was purposively chosen. All schools were single intake and one class each from year 2 (age 6–7), year 4 (age 8–9) and year 6 (age 10–11) were selected for participation. Ethical approval for the study using opt-out parental consent was obtained from a higher education institutional research ethics committee (17/PBH/008). Written informed consent was obtained from all head teachers, information letters that included an option to withdraw children from the study were sent home to parents via the school and all children completed an assent form before participating. 

### 2.2. Data Collection

Data was collected via a workbook which included a Draw and Write exercise (D&W) and a survey and peer discussion groups in either English (*n* = 7 schools) or Welsh (*n* = 1 school). The response rate range (based on the number of workbooks completed, and number of letters sent out to parents for each school) was 75.6–100%. Tool development was guided by previous research [11,14,45,46] and piloted to ensure age appropriateness. The workbook was administered by 4 experienced female researchers and 2 Welsh speaking female research assistants to the whole class for year 4 and year 6 and in small groups for year 2. D&W is a widely used class-based tool for capturing rich insightful data from children in a short timescale [47,48]. Children were instructed by a researcher to draw a person who smokes and describe why they smoke, how they feel and what they would see and smell if standing nearby. This was repeated for a person using e-cigarettes. Children were then instructed to complete the survey, which collected basic demographic information (sex, age), family smoking and vaping behaviours, knowledge of and attitudes/beliefs about electronic and tobacco cigarettes, and future intentions to smoke or vape. Lastly, small group mixed-sex peer discussions were conducted with a subsample of children (*n* = 96). Two boys and two girls from each participating class were purposively selected by the teacher on the basis they would be confident and comfortable speaking with researchers outside the classroom [45]. To assess knowledge of and attitudes toward smoking and vaping, perceptions of health risks and social norms, participants were asked to discuss what they knew about electronic and tobacco cigarettes, who they thought vaped and smoked and why, the consequences of vaping and smoking and the influence of flavours on vaping behaviour. A series of photographs depicting tobacco and e-cigarettes, e-liquids and a range of people vaping, and smoking were used as elicitation devices to help prompt discussion with the children [49]. 

### 2.3. Data Analysis

Data from the three tools were analysed independently and findings triangulated, which is the process of examining data from multiple sources to corroborate findings and enhance the credibility and validity of the results. For D&W, an iterative qualitative coding framework was developed from the responses [34]. The children’s written responses were coded by one researcher, refined and then combined into content categories. Simple frequency counts were used and themes in the data were identified. A child’s response was only counted once in each category but could be coded to several categories, in the event that the answer had multiple responses. Children’s drawings were not coded but used to illustrate typical themes emerging in the data. Another researcher reviewed the coding system to aid the credibility and trustworthiness of the analysis, any anomalies were discussed, and a final decision was jointly made. Survey data were entered, cleaned and analysed in SPSS Statistics for Windows, v23 (IBM Corp., Armonk, NY, USA). Analyses used frequencies, descriptive statistics and chi-squared tests. Peer discussions were recorded using a digital audio device, transcribed verbatim and imported into QSR NVivio 11 (QSR International Pty Ltd. Version 11, 2012, Melbourne, Australia) to assist with coding and analysis. The native Welsh speaking research assistants transcribed the Welsh peer discussions and checked each other’s work for accuracy. Thematic analysis [50] carried out by one researcher and cross-checked by another was both inductive, with themes emerging from the qualitative data and deductive, informed by existing literature and the topics covered in the peer discussion. Transcripts were read and open coded line by line. Codes were then grouped into categories and examined for salient themes [50]. Saturation was considered to be reached as no new codes were identified in the final transcripts analysed.

## 3. Results

### 3.1. Participant Characteristics

Similar numbers of children were recruited from each year group: aged 6–7 years (33.3%); 8–9 (37.4%) and 10–11 (29.3%) and 27.8% reported that they spoke the Welsh language at home. 

Two schools were in urban areas, five were rural and one semi-rural. Two schools were in the most deprived and two schools in the least deprived deprivation category [51] (Table 1).

### 3.2. Awareness of E-Cigarettes

Children across the 3-year groups demonstrated a general awareness of e-cigarettes. The vast majority (94.9%) could distinguish pictures of e-cigarettes from tobacco cigarettes in the survey (year 2, 89%; year 4, 96.7%; year 6, 100%: *p* < 0.001). Most thought electronic and tobacco cigarettes looked different from each other (93.3%) and that the internal components were different (82.5%). Around half reported they smell different (51.1%) and the smoke was different (48.0%) (Table 2).

Awareness of e-cigarettes was evident in the peer discussions as well. Children across all 3-year groups were able to discern the difference between tobacco and e-cigarettes from a series of photographs and discuss them using appropriate terminology such as ‘e-cigarettes’, ‘e-cigs’, ‘electric fags’ and ‘vapes’ and acknowledged they were different from tobacco cigarettes.

*”They look like different from normal fags.”*

(Male, Age 7, School 4)

*“They [e-cigarettes] don’t have any tobacco in.”*

(Male, Aged 9, School 7)

Younger children were of the opinion that e-cigarettes were comprised of ‘metal and oil’ or ‘plastic’ and ‘glass’. Some believed they contained ‘chemicals’ and considered them to be more durable and not disposable like tobacco cigarettes:

*“They might be stronger longer and you don’t throw it away.”*

(Female, Age 7, School 1)

*“I think they’re better for you, you can charge them back up, you don’t have to throw them out and that could start a fire.”*

(Male, age 7, School 6)

Older children exposed to people who vape had a more nuanced understanding of e-cigarettes and were able to better discuss their composition, how they are used and the differing nicotine levels.

*“It’s like a vape, you put liquid in them, because my stepdad has one, you put liquid in them and then that burns out. It’s got nicotine in it, some of them have.”*

(Male, Age 11, School 4)

*“E-cigarettes can have nicotine and also not because my dad is down to zero nicotine now, he doesn’t use nicotine in his vape at all and you can customise the flavour and that, he prefers peach.”*

(Male, Age 11, School 5)

In the survey, a higher proportion of children thought tobacco cigarettes were used more (59.9%) and were easier to purchase (57.8%), than e-cigarettes. 73.7% reported seeing tobacco cigarettes more often than e-cigarettes (14.5%). Over six in ten (61.8%) also reported that e-cigarettes were safer to use than tobacco cigarettes (Table 2). This was evidenced in the qualitative findings with some of the younger children in particular, highlighting that e-cigarettes did not need to be lit and therefore would not be a fire hazard. Several of the older children brought up the fact that e-cigarettes can explode ‘in ‘your pocket’ or ‘in your face’ and stressed the importance of getting ‘a proper one from special shops’, for safety purposes.

*“I think the electronic one is more safer than the other [tobacco] one because if we blow it too hard and we drop it and it is still flaming it might cause a forest fire or a house fire.”*

(Male, Aged 7, School 3)

*“With the other ones [tobacco cigarettes] if you light it and drop it on the carpet it can cause a fire.”*

(Female, Aged 9, School 5)

Flavours featured widely in children’s views of e-cigarettes. Of the 401 D&W responses that describe what children see and smell when near e-cigarettes, 22.9% (*n* = 94) specifically mentioned ‘sweet, scented smoke’, ‘nice smells’ and fruit flavours (year 2, *n* = 23; year 4, *n* = 24, year 6, *n* = 47) (Table 3).

In the peer discussions, awareness that e-cigarettes ‘got fruit flavours inside of it’ was commonplace and there was a general consensus across all age groups that sweet and fruit flavoured e-liquids were more likely to appeal to young people and could ‘encourage’ them to vape.

*“The young ones might be encouraged to use it, the electronic one, because they love fruit and they might not know that it is really unhealthy”*

(Female, Aged 7, School 3)

*“I think that the younger people will want to do it because with all the different flavours, they just want to try them”*

(Female, Aged 9, School 1)

*“(…) to encourage younger people like 15-year olds because of the flavours like cherry, chocolate...”*

(Female, Aged 11, School 6)

A couple of the older children also considered the aesthetic appeal of e-liquid flavours and their commercial potential:

*“(…) because their breath doesn’t smell”*

(Female, aged 11, School 7)

*“I think they’re trying to make it more appealing to children by making the like bubble gum flavoured and candy flavoured… so they have more buyers.”*

(Male, aged 11, School 1)

In peer discussions, many children were aware of a legal age of purchase for tobacco and e-cigarettes, although the majority were unsure of the exact age (often stating 16 rather than 18 years). Many were uncertain about where to access e-cigarettes. Those with relatives who vaped were better able to identify places of purchase including specialist shops, supermarkets and online. There was some misunderstanding of the legal consequences of purchasing tobacco and e-cigarettes underage, with some believing that children or their parents could go to prison if they were found to be smoking or vaping.

*“Anyone over the age of sixteen [can buy them].”*

(Male, Aged 7, School 2)

*“You need to get a proper one [e-cigarette] from special shops. My mum has a proper one.”*

(Female, Aged 11, School 6)

Some comprehension of addiction and, to a lesser extent the role of nicotine was evident, primarily amongst the older children. Survey responses identified that two-thirds of the children thought it would be hard to stop smoking tobacco cigarettes once started whilst nearly half (46.9%) felt the same for e-cigarettes. There were significant differences in responses across year groups for both tobacco (year 2, 66.5%; year 4, 66.8%; year 6, 68.3%: *p* < 0.001) and electronic cigarettes (year 2, 53.7%; year 4, 48.1%; year 6, 38.0%: *p* < 0.001).

In the D&W exercise, the word addicted was mentioned 103 times, almost exclusively regarding how tobacco smokers feel and why they smoke tobacco cigarettes. Addiction in relation to tobacco smoking was mentioned in all year 6 peer discussions but never with reference to e-cigarettes. Nicotine was referred to less frequently by older children when discussing either electronic or tobacco cigarettes. Younger children almost never used the terms addiction or nicotine when discussing smoking or vaping, but discussions did convey some understanding of the concepts.

*“If people still want to smoke…but they know the tobacco is affecting their lungs they just use those [e-cigarettes] cause it’s got the nicotine in.”*

(Female, Aged 7, School 6)

A couple of the older children made the connection between the two:

*“They can also be really bad as well because say they had a flavour in them and they had nicotine then because the flavour could be addictive and then there is nicotine in it so could be then it could be the same as a normal cigarette.”*

(Male, age 11, School 5)

A few of the children across all the discussion groups knew that the amount of nicotine in e-cigarettes was variable and could be altered to aid smoking cessation. These children often had family members who vaped.

*“And with them [e-cigarettes]…You can cut down….you can get ones with no nicotine at all.”*

(Male, Age 9, School 2)

*“My father stopped smoking about 3 years ago now, he’s been on vapes since then. He has been lowering the nicotine and he is down to zero now.”*

(Male, Age 11, School 5)

### 3.3. Health Harms of E-Cigarettes 

In general, children had little understanding of any health harms associated with e-cigarettes, often associating vaping and smoking with similar levels and types of harms. D&W responses for perceived health harms (n=385) ranged from e-cigarettes being as bad for health as tobacco cigarettes (*n* = 17 (4.4%); year 2, *n* = 4; year 4, *n* = 5; year 6, *n* = 8) and/or causing death (*n* = 14 (3.6%); year 2, *n* = 6; year 4, *n* = 4; year 6, *n* = 4) to e-cigarettes being healthier and less harmful than tobacco cigarettes (*n* = 28 (7.3%); year 2, *n* = 10; year 4, *n* = 12; year 6, *n* = 6) or not harmful at all (*n* = 26 (6.8%); year 2,

*n* = 8; year 4, *n* = 12; year 6, *n* = 6). Three times as many older children did not know of any health harms associated with e-cigarettes (*n* = 116 (30.1%) year 2, *n* = 22; year 4, *n* = 32; year 6, *n* = 62) (Table 3).

Similar misperceptions emerged in the peer discussion groups. Younger children in particular, considered e-cigarettes to be healthier and less harmful than tobacco cigarettes, primarily because they contained fruit flavours:

*“It’s strawberry flavoured and strawberries are healthy.”*

(Female, Aged 7, School 1)

*“I think the electronic one [is healthier] because it has fruits in.”*

(Female, Aged 7, School 3)

One older child (age 11) felt ‘safer’ breathing in flavoured rather than cigarette smoke:

*“I know someone that had one once and it was cherry flavour and every time they puffed it out it smelled really nice, so it made me feel a bit better and safer that I’m not breathing in all the bad things.”*

(Girl, Age 11, School 6)

Unsurprisingly, older children had a better grasp of the health consequences of tobacco smoking and whilst many were unsure how e-cigarettes impacted on health, they surmised they would be better than or different to tobacco cigarettes, mainly because they of their composition:

*“There is not tobacco in them so they can’t harm you as much.”*

(Male, Age 11, School 7)

*“They are ever so slightly better because they have no tar and stuff”*

(Male, Age 11, School 6)

*“After looking at the fruit flavours, I think you would have different damage to the body because I think tobacco will have more damage to the body like lung cancer”*

(Male, Age 11, School 3)

There was also some appreciation by older children that potential health harms of e-cigarettes are still unknown and therefore caution is warranted:

*“I think people get a bit like ‘Oh I want to try that’ but they don’t actually know the harms they can do to you.”*

(Female, Age 11, School 6)

*“In about 2 years’ time we could find out that they’re [e- cigarettes] even worse than the other ones but we don’t know yet.”*

(Male, Aged 11, School 1)

The notion that e-cigarettes were healthier than tobacco cigarettes prevailed throughout the study although e-cigarettes were still considered to be more harmful than not smoking or vaping at all. Survey results showed that more than half believed that tobacco cigarettes were worse for smoker’s lungs (59.6%) and worse for other people’s lungs (55.4%) compared to e-cigarettes. This increased with age from 46% of children aged 7 years to 72.7% aged 11. More children (74.6%) thought smoking was never a good thing to do compared to those who thought using an e-cigarette was never a good thing to do (57.5%). Children were less likely to feel that using an e-cigarette was never a good thing to do if they lived with somebody who used them (30.9%) compared to not living with someone using e-cigarettes (65.2% *p* < 0.001) (Table 2). This view also featured in the peer discussions. Whilst children generally did not think it was okay to use e-cigarettes, some felt that people who wanted to be healthy would be more likely use e-cigarettes than smoke tobacco cigarettes.

*“Well they don’t damage your lungs like tobacco ones because you don’t have the ash in it.”*

(Male, Aged 7, School 5)

*“People class them as like a healthier way of using cigarettes, people think they are better but they’re not.”*

(Female, Aged 11, School 4)

### 3.4. Motivations for Vaping 

When asked why people vape, over a quarter of D&W responses (27.6% (*n* = 102); year 2, *n* = 20; year 4, *n* = 38; year 6, *n* = 44) indicated that e-cigarettes are used primarily to stop smoking tobacco cigarettes (Table 3). Quitting was the main reason given in the peer discussions as well and an awareness of the role e-cigarettes play in smoking cessation was highlighted by some.

*“If they have the real fags, they are bad, and they damage your lungs. If you have them ones [e-cigarettes], they stop them, they stop the real fags and then you won’t smoke them.”*

(Male, Age 7, School 4)

*“Because they want to stop smoking. They use it as bit of a jump. That’s what they are intended for I think. They are intended to be a bit of a steppingstone to stopping.”*

(Male, Age 11, School 6)

*“People who smoke normal tobacco, they wanted to quit so they started smoking the electronic.”*

(Male, Aged 11, School 3)

Notably, children who had family members who vaped or smoked were better able to discuss how using them helped parents and relatives to quit smoking in peer discussions.

*“He [dad] has mostly stopped using them [tobacco cigarettes] and just uses those [e- cigarettes].”*

(Male, Aged 7, School 5)

*“My step-dad has one, you put liquid in them and then that burns out. It’s got nicotine in it, some of them have.”*

(Male, Aged 11, School 4)

Other reasons for using e-cigarettes mentioned in D&W responses (*n* = 370) included: because they are better than smoking 12% (*n* = 44), because they are healthier than tobacco cigarettes 11% (n=39), because it is enjoyable and fun (11%), to look cool and popular (10%) or because their friends do (2%). In peer discussions some suggested that teenagers use e-cigarettes to look cool and fit in with peer groups, but these social motivations were more widely associated with tobacco cigarettes.

*“They [e-cigarettes] look safe and you want it to look cool and you look more cool with them.”*

(Female, Age 9, School 6)

### 3.5. Future Intentions to Vape

Few children expressed any intention to use e-cigarettes or smoke tobacco cigarettes when older (Table 2). Of the minority reporting future intentions, slightly more thought they would vape (3.9%) rather than smoke (1.8%). Intention to smoke was significantly more likely if the child lived with someone who smoked (*p* = 0.02), and intention to vape was significantly more likely if they lived with an e-cigarette user (*p* < 0.001) (Table 2). Older children were significantly less likely to say they would vape when older (age 10–11, 1.4%) than younger children (age 6–7, 6.7%), *p* < 0.001).

The majority of peer discussion participants thought that they would not smoke tobacco or use e-cigarettes in the future although a few older children did say they might try e-cigarettes when older because they perceived them to be less harmful and taste better than tobacco cigarettes.

*“If I’m talking about now, I have never actually smoked so I don’t know what it’s like so when I’m 18 I might think ‘oh what’s it like?’ and then you can’t stop smoking because it’s addictive.”*

(Male, Aged 11, School 1)

### 3.6. Acceptability of Vaping and Smoking

In the survey, almost half of the children reported it was okay for grown-ups to use e-cigarettes (49.6%) or tobacco cigarettes (46.2%). Results were age related, with acceptability decreasing with age for both tobacco smoking in adults (*p* < 0.001) and vaping in adults (*p* = 0.024). Acceptability in adults was influenced by exposure to e-cigarettes in the home. Over a fifth (22.4%) indicated that somebody who lives in their household used e-cigarettes and 32.3% had somebody in their household who smoked tobacco (Table 2). Children who lived with an e-cigarette user were more likely to report that it was ok for grown-ups to use e-cigarettes (63.0%), compared with those who did not live with someone who used e-cigarettes (45.5%) (*p* = 0.03) (Table 2).

Subtle familial influences were noted in peer discussion data. Findings suggest that children who had family members that were e-cigarette users or smokers were much more knowledgeable about the products and could discuss how they were used and where they could be purchased. These children were also able to comment more broadly on the variety of fruit flavored liquids available and the use of e-cigarettes to stop smoking. They tended to be less negative about people using them compared to those who had limited exposure. Some understanding of role modelling was evident as well.

*“Also, because sometimes if your mum or dad smokes they influence you because you’re trying to be like your mum or dad or grownups—so if you see somebody who inspires you smoking you kind of want to do that.”*

(Male, Age 9, School 8)

Acceptability of e-cigarette use in children was very low. Six (1.2%) participants thought it was okay for children their age to use e-cigarettes and only 1 (0.2%) to smoke tobacco cigarettes. There were no significant differences in acceptability of e-cigarette use by gender or age. It was also low for children who lived with e-cigarette users (1.8%) and those not living with e-cigarette users (1.0%) but this difference was statistically significant (*p* = 0.005) (Table 2).

Many of the children in peer discussions thought it was more acceptable for adults to use electronic and tobacco cigarettes than children because of the legal age restriction. Furthermore, there was a perception that adults were better able to make decisions about behaviours that had potential risk. Some children suggested that the smoke from tobacco cigarettes was more harmful to children’s bodies because they were still developing.

*“ [Older people] they will be older then and their lungs won’t be damaged that much [compared to younger people].”*

(Male, Aged 7, School 5)

*“Maybe your veins go stronger [when you are older], so maybe your veins can handle it.”*

(Male, Aged 9, School 1)

## 4. Discussion

As one of the first studies to investigate e-cigarettes in the context of childhood, this research contributes unique and important insights into primary schoolchildren’s perceptions of vaping, confirming that those aged 11 years and younger are already assimilating knowledge about and forming perceptions of vaping. Consistent with previous research on adolescents, there was general awareness of e-cigarettes and most could differentiate between electronic and tobacco cigarettes. [2,52,53]. Viewing smoking and vaping as distinctly different is important to mitigate against the re-normalisation of smoking behaviour [40,54,55].

In line with previous studies on older populations [11,39,56,57] children had awareness of flavoured e-liquids and believed that sweeter ones might encourage young people to vape. A few of the youngest children believed the fruits in flavourings made them healthier. Such findings are noteworthy given concerns about the attraction of flavoured e-cigarettes to young people and recent calls to prohibit them [25,33]. Further research is needed to understand the potential appeal of flavoured e-cigarettes to youth and assess if further regulation is warranted.

Our study found that many children were aware of the role e-cigarettes play in smoking cessation, particularly if a family member vaped. This is unsurprising given that stopping smoking is often cited as the primary reason for vaping [58]. Whilst the evidence for the efficacy of e-cigarettes to help smokers quit is inconclusive [18,23,24], use continues to grow with 3.6 million adults (7.1%) currently vaping in GB [58]. It is important that children continue to perceive e-cigarettes as a pathway to smoking reduction and cessation for adult smokers rather than a pleasurable activity in its own right, which may encourage experimentation and initiation. As such, health messaging efforts should seek to ‘normalise quitting behaviour’ [40] in addition to preventing uptake of smoking and vaping altogether. 

One prevailing perception that emerged from the study was the belief that e-cigarettes were ‘healthier’ than tobacco cigarettes. Perceiving e-cigarettes as ‘healthy’ rather than less harmful potentially masks the fact that their use is not without risk. Given that previous research with older school children (11–16 years) in the UK concluded that never smokers who considered e-cigarettes to be a ‘safer option’ could be at risk of later tobacco use [8], health promotion efforts need to reframe children’s perceptions of e-cigarettes. Reinforcing the message based on current evidence, that vaping is considered to be ‘less harmful’ rather than ‘healthier’ than smoking and highlighting the associated risks of e-cigarette experimentation, including the potential for tobacco initiation.

Relative to smoking, most children had little or no understanding of e-cigarette health harms with over half associating vaping with the same health consequences as smoking. Whilst not unexpected given the children’s developmental ages, it does reflect a wider trend [59]. The proportion of 11-18-year-olds in GB who believe e-cigarettes are equally as harmful as tobacco cigarettes has increased over the past 4 years from 21% in 2015 to 30% in 2019 [2]. Some awareness of addiction was evident, but this was largely related to tobacco smoking. Similar to previous research [8], few children understood the substantive role of nicotine, particularly in relation to e-cigarettes. There was also uncertainty about the legal age of purchase for both tobacco and e-cigarettes. The lack of knowledge and prevailing misperceptions provide a strong rationale for the inclusion of e-cigarette education into the current drug education curriculum in primary schools, to help develop understanding, address uncertainties, dispel misconceptions and discourage future uptake. 

Study findings demonstrated that exposure to e-cigarettes influences children’s perceptions. Children who lived with someone who smoked or vaped appeared to have greater knowledge and understanding of the products, were more accepting of electronic and tobacco cigarettes and more likely to express intention to vape or smoke as grownups. Given the influence of the family on children’s perceptions of electronic and tobacco cigarettes and knowing that children’s future behaviour is related to adult’s current role modelling behaviour [60,61] familial involvement in any health promotion measures to prevent experimentation and uptake of vaping is imperative. Parents who use electronic and tobacco cigarettes need to be made aware that through the influence of example, their behavior may prompt their children to vape or smoke in the future. To mitigate against this risk, parents should be educated and encouraged to stop smoking. Parents who vape to reduce smoking related harm should discuss their reasons for doing so with their children, to avoid any misperceptions around use [40].

Consistent with previous tobacco research [43] children generally had negative perceptions of both vaping and smoking, and most did not intend to use electronic or tobacco cigarettes when older. Of concern was the finding that almost all viewed vaping and smoking as inappropriate for children their own age but almost half considered it to be acceptable adult behaviour. Given that positive attitudes toward smoking may predict intentions to smoke in the future and later smoking behaviour [62], further research is needed to understand why this acceptability persists despite extensive tobacco control efforts and pervasive anti-smoking social norms. The extent to which vaping leads to future smoking also requires further investigation.

### Study Limitations

Our study was based a small sample of eight Welsh schools, purposively selected for maximum variation, and therefore findings cannot be generalised to all children in Wales. As a school based mixed methods study conducted in a classroom setting, children may have influenced each other’s responses, and their participation in earlier stages may have influenced responses in the later stages.

## 5. Conclusions

This study provides unique insights into Welsh primary school children’s perceptions of e-cigarettes and highlights the importance of exploring younger children’s understanding of vaping relative to smoking. Whilst children in the study had general awareness of e-cigarettes, understanding of health harms was limited, characterised by misconception and uncertainty. The findings also demonstrate how primary school children contextualise their understanding of e-cigarettes based on their own experiences and existing knowledge of tobacco cigarettes. 

Primary school children represent an important cohort for primary prevention. Understanding how they perceive e-cigarettes before experimentation is essential to inform current and future tobacco control strategies and prevent uptake in children and young people. Study findings should prompt policymakers, practitioners, educators and parents to consider the impact of e-cigarette use on primary schoolchildren and work toward minimising potential risks via appropriate policy responses and educational practice.

## Figures and Tables

**Table 1:** Participant and school information.

Variable	Category	*n*	%
**Gender**	Boy	236	47.8
	Girl	258	52.2
**Year group (age in years)**	Year 2 (6–7)	165	33.3
	Year 4 (8–9)	185	37.4
	Year 6 (10–11)	145	29.3
**Speak Welsh at home**	Yes	135	27.8
	No	350	72.2
**School**	1	77	15.5
	2	68	13.7
	3	61	12.2
	4	72	14.5
	5	68	13.7
	6	20	4.0
	7	81	16.3
	8	51	10.2

**Table 2:** Survey findings: children’s knowledge, perceptions and intention to use electronic and tobacco cigarettes (*n* = 494).

Variable	Category	%			
**Differentiating electronic and tobacco cigarettes on……**		Same	Different	Not Sure	
	Look	3.7	93.3	3.1	
	Smell	27.0	51.1	21.9	
	Taste	11.6	46.1	42.3	
	Cost	5.3	67.8	26.9	
	Inside	6.0	82.5	11.5	
	Smoke	32.2	48.0	19.8	
**Perceptions of electronic and tobacco cigarettes:** **Which do you…**		Electronic Cigarette	Tobacco Cigarette	Both the same	Don’t know
	See more often	14.5	73.7	8.0	3.9
	Think more people use	18.2	59.9	13.3	8.6
	Think is safer to use	61.8	15.7	6.5	15.9
	Think it is easier to buy	21.6	57.8	7.2	12.9
**Intention to use:**		Yes	No	Maybe/don’t know	*p*-Value
**Electronic cigarettes**	Lives with user	7.3	55.0	37.6	0.02
	Does not live with user	2.9	78.6	18.5	
**Tobacco cigarettes**	Lives with smoker	3.1	75.5	21.4	<0.001
	Does not live with smoker	1.2	88.7	10.1	
**Acceptability of Adult Use:**		Yes	No	Maybe/don’t know	*p*-Value
**Electronic cigarettes**	Lives with user	63.0	14.8	22.2	0.03
	Does not live with user	45.5	27.9	26.6	
**Tobacco cigarettes**	Lives with smoker	51.3	27.2	21.5	0.423
	Does not live with smoker	43.7	31.1	25.2	
**Acceptability of Child Use:**		Yes	No	Maybe/don’t know	*p*-Value
**Electronic cigarettes**	Lives with user	1.8	84.4	13.8	0.005
	Does not live with user	1.0	94.2	4.8	
**Tobacco cigarettes**	Lives with smoker	0.6	97.5	1.9	0.502
	Does not live with smoker	0.0	98.5	1.5	

**Table 3:** Draw and Write Responses for e-cigarette users.

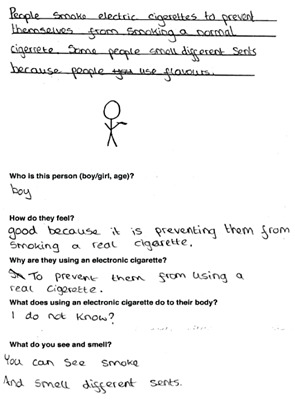	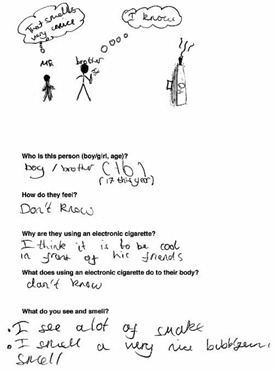
Female, Aged 11, School 5	Male, Aged 11, School 4
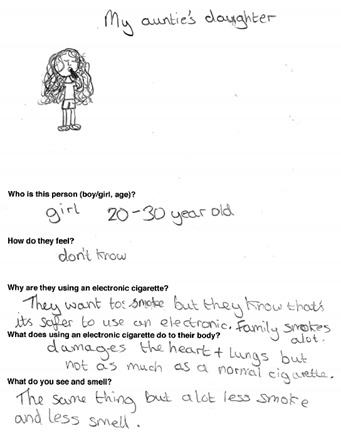	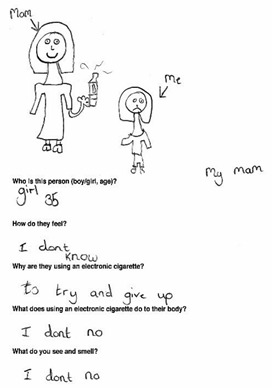
Female, Aged 11, School 2	Female, Aged 9, School 4
